# Halloysite Nanotubes: Interfacial Properties and Applications in Cultural Heritage

**DOI:** 10.1021/acs.langmuir.0c00573

**Published:** 2020-03-23

**Authors:** Giuseppe Cavallaro, Stefana Milioto, Giuseppe Lazzara

**Affiliations:** Dipartimento di Fisica e Chimica, Università degli Studi di Palermo, Viale delle Scienze, pad. 17, 90128 Palermo, Italy; Consorzio Interuniversitario Nazionale per la Scienza e Tecnologia dei Materiali, INSTM, Via G. Giusti 9, I-50121 Firenze, Italy

## Abstract

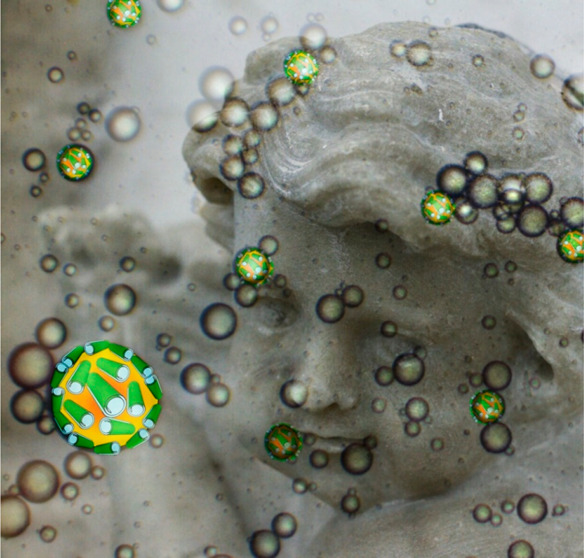

The peculiar surfaces of halloysite nanotubes and their biocompatibility are attracting the interest of researchers based on the wide range of attainable applications. The large aspect ratio of this nanotubular material ensures promising properties as a reinforcing agent in polymeric matrixes, such as cellulose and its derivatives, that entail strengthening due to, for instance, aging-induced degradation. The halloysite cavity has a suitable size for hosting a large variety of active species such as deacidifying (calcium hydroxide) and flame retardant agents (fluorinated surfactants) for a controlled and sustained release relevant to the conservation of cultural heritage. Additionally, anionic surfactants can be selectively adsorbed at the inner surface generating inorganic micelles able to solubilize hydrophobic species in a controlled cleaning protocol. We briefly discuss how the natural halloysite nanotubes can be supportive in various conservation processes of cultural heritage and present an outlook for future perspectives.

## Introduction

Halloysite nanotubes are natural nanoclays with attractive surface chemistry.^1−4^ Natural deposits are located worldwide, the largest ones being in New Zealand and Utah (U.S.). It can be considered to be a safe and biocompatible nanomaterial because it was demonstrated to have low toxicity toward worms, microorganisms, and rats.^5−8^

**Figure 1:**
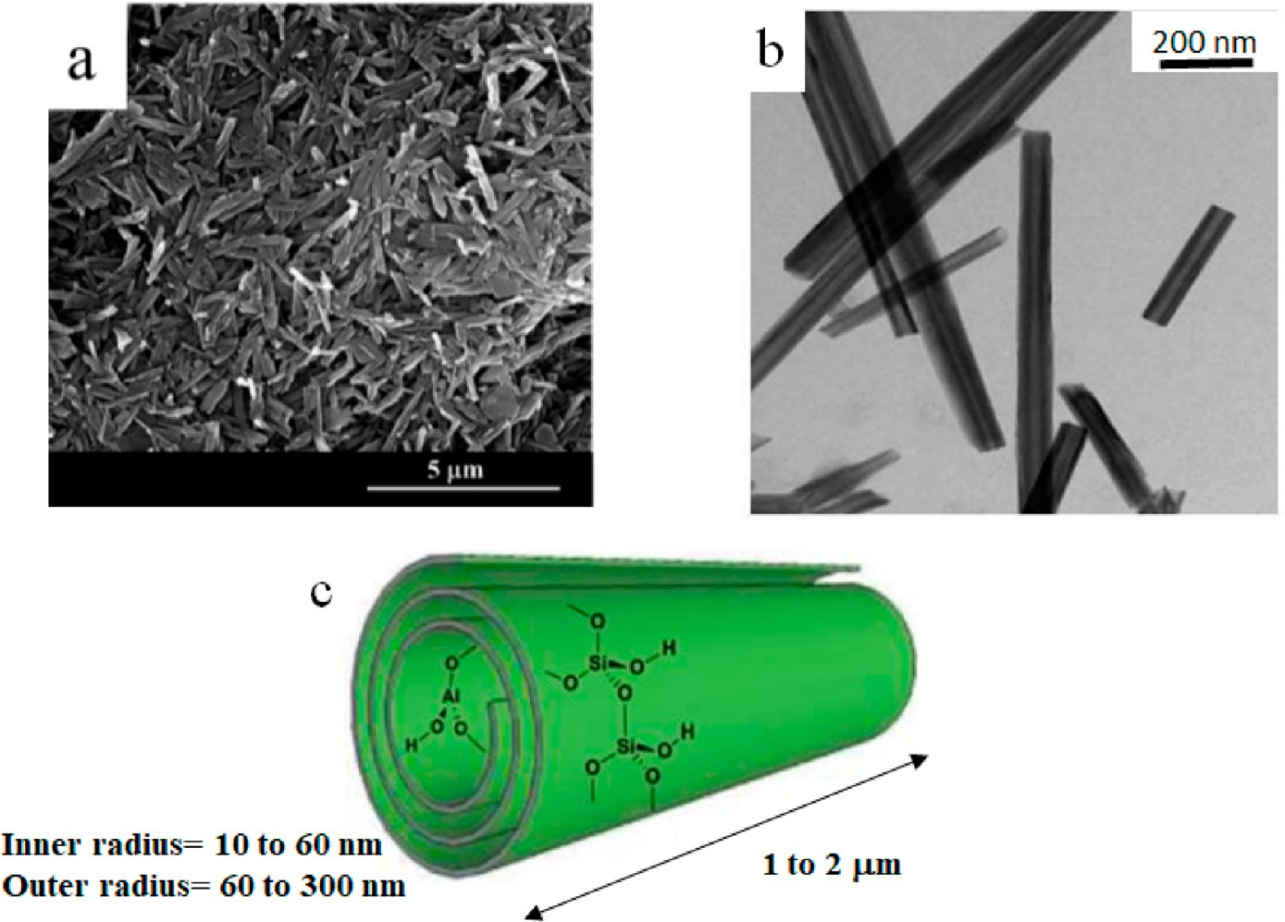
(a) SEM and (b) TEM images of halloysite nanotubes. (c) Schematic representation of the spiral-like morphology of halloysite. Adapted with permission from refs (24) and (53) for (a) and (b), respectively.

The typical sizes, polydispersity, and mineral purity of halloysite nanotubes (HNTs) are affected by their specific geological origin.^9−11^ In general, the hollow tubular shape, formed by aluminosilicate layers with spiral-like morphology, is characterized by a length in the micrometer range, while the external and internal diameters are between 60–300 and 10–60 nm, respectively (Figure 1). The interlayer distance is 1 or 0.6 nm depending on the hydration state of halloysite. In fact, the unitary cell formula is Al_2_Si_2_O_5_(OH)_4_·*n*H_2_O, similar to the most common kaolinite except for the presence of the water molecules (typically 2) that are hosted between the adjacent clay layers. Although simulations demonstrated that very minimal distortions are expected in the rolled sheets that distinguish the halloysite morphology,^12^ the packing disorder and the interlayer water molecules might induce the transformation of kaolinite to clay nanotubes.^1,13^ In terms of applications, the main interesting features of halloysite are (i) surface reactivity, (ii) a hollow cavity, (iii) easy dispersibility and stability in solvent media, and (iv) long aspect ratios,which are detailed below.

Several examples of covalent^14−19^ and weak or electrostatic interactions^20−26^ are reported for the selective modification of HNTs inside or at the outer surface. The interaction with amphiphilic molecules endowed the formation of inorganic micelles, with the aim of covalent binding to alumina groups of the HNT cavity (octadecylphosphonic acid^16^ and dopamine derivatives^27,28^) or ion-exchange capability for selective binding with the halloysite positive lumen^21,23,29,30^ or negative outer surface^26^ reported. These strategies endow the preparation of functional nanocontainers for loading and the sustained release of active species.

The described features, in particular, the presence of a hollow cavity, are certainly strategic in the research devoted to new drug delivery systems; therefore, most of the reported applications are in this field as summarized in the following text.

Temperature-responsive nanocarriers were prepared by the selective functionalization of halloysite with poly(*N*-isopropylacrylamide) for the controlled delivery of drugs.^15^ Also, the design of biohybrid halloysite-based materials was proposed for health applications such as antimicrobial patches, tissue engineering,^31−34^ and drug-releasing systems.^35−38^

Interestingly, the same general principle was exploited for the encapsulation of corrosion inhibitors, with the aim of generating a self-healing protection layer on metal surfaces,^39−42^ and of antioxidant species for food packaging.^43,44^ Due to the high specific surface and easy dispersion in solvents compared to other clays, halloysite nanotubes also showed interesting catalytic activity in combination with metal nanoparticles.^45−49^

The large aspect ratio of halloysite nanoparticles, similar to other nanotubular materials and nanofibers, encouraged its use as a filler for plastic and bioplastic materials to enhance mechanical and transport properties.^10,50−52^

More recently, halloysite nanotubes are emerging in the field of conservation science for cultural heritage, and here we present the advances in this domain by our group and the perspectives that are open for further applications.

## Role of Interfaces and Cavity in Halloysite Nanotubes

The surface chemistry of halloysite nanotubes is very attractive because of the differences between the inner and outer surfaces: the external surface consists of Si–O–Si, while the inner one is a gibbsite-like array of Al–OH groups. This fact allows for targeted modifications using selective functionalization and a peculiar charge separation within the nanotube. Moreover, the different acid–base behaviors of alumina and silica are responsible for a positively charged lumen and a negatively charged outer surface in a wide pH interval (from 3 to 8).^6^

The crystalline phase behavior of clay nanotube aqueous dispersions depends on the concentration, the pH, and the presence of electrolytes.^54,55^ The ordering of the nanotubes can be achieved via droplet casting in the presence of anionic polystyrenesulfonate or in a capillary system.^33,56−59^ On the basis of geometrical consideration, the typical overlapping concentration, namely, the concentration at which the nanotubes start overlapping because they are at the contact distance, for halloysite nanotubes is at about 6 to 8 wt %.^4^ This threshold represents the limit for the concentration in the so-called sedimentation volume that is a stable concentrated phase of halloysite dispersed in water.^4,23^

Additionally, supramolecular interactions between ionic molecules and HNT surfaces influence the aqueous colloidal stability of the nanotubes.^60^

The halloysite colloidal stability, sedimentation, and dispersibility in water are key features for any application. The presence of anionic and nonionic polymers^20,61^ enhances the dispersion of the nanotubes as a consequence of the increase in the net negative surface charge or due to a steric barrier that opposes nanotube clustering. On the other hand, adding sodium chloride generates a screening of the electrostatic repulsion and enhances the nanotubes’ sedimentation process.^21,60^

Beside the peculiar surface properties, halloysite has a cavity that represents ca. 10% of the volume of the nanotube, and it is in the nanometer size range. On this basis, it is suitable to confine and accumulate small molecules and even macromolecules such as proteins.

A liquid that is confined in the halloysite lumen provides different physicochemical properties from those of the bulk solvent. This aspect is relevant to generating peculiar nanoreactors, and it plays a major role in the strategy to load molecules from solutions.

**Figure 2:**
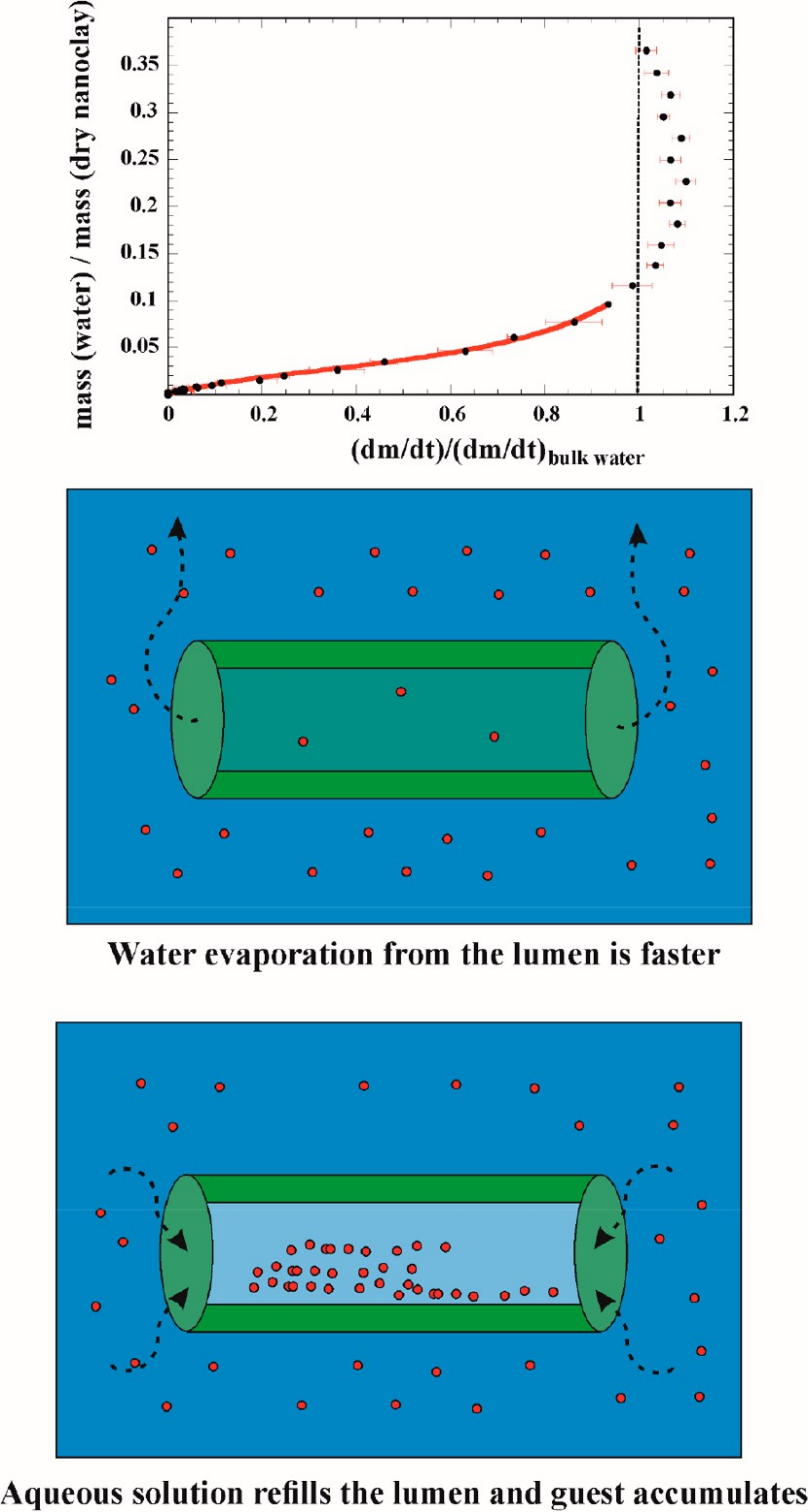
(Top) Mass loss rates for the nanoclay aqueous dispersions normalized for pure water evaporation as a function of the mass ratio between water and dry nanoclay. (Bottom) Schematic representation of the driving force for the loading process of halloysite lumen. Adapted with permission from ref (63).

The encapsulation of liquids inside carbon nanotubes leads to an increase in the water activity.^62^ Then, similar observations were made for water confined in halloysite nanotubes.^63^ Hence, we demonstrated the faster evaporation rate of confined water within the inner lumen of halloysite (Figure 2), and this finding was shown to be correlated to the driving mechanism of nanotube filling and the accumulation of solute in the nanotube lumen. In particular, Knudsen thermogravimetry was used under isothermal conditions on concentrated aqueous dispersions of halloysite (ca. 30 wt %). Due to the peculiar cell design, the isothermal water evaporation from wet nanoclays is measured and the mass loss time derivative (that corresponds to the evaporation velocity (d*m*/d*t*)*_n_*) is proportional to the water activity in the sample after normalization for the bulk water evaporation rate ((d*m*/d*t*)_w_).^63^ These experiments clearly evidenced (Figure 2) that the water from the lumen has an evaporation rate larger than that of bulk water as (d*m*/d*t*)*_n_*/(d*m*/d*t*)_w_ > 1 for mass ratios of between 0.12 and 0.3 which corresponds to the predictable filling capacity based on geometric considerations and densities.^63^

Due to its higher vapor pressure, the water confined inside the nanotubes’ cavity can evaporate faster than the bulk water. The difference of the water evaporation rate represents the main driving force for the filling of HNT cavity through aqueous dispersions. Price et al. reported that by subjecting the drug/clay dispersion to vacuum pumping steps, the drug loading efficiency is greatly enhanced for comparison with encapsulation protocols carried out without any particular pressure control.^64^ The scheme in Figure 2 reports the driving force and physicochemical explanation for the payload efficient accumulation in the halloysite lumen.

## Applications in the Conservation of Cultural Heritage

In the last few years, green nanomaterials have attracted a growing interest by the scientific community in order to develop sustainable protocols for curing cultural heritage. In this regard, several other clay nanoparticles (such as laponite, montmorillonite, and sepiolite) have been proposed for the restoration and conservation of artworks. In particular, they are typically used as thickener agents to prepare formulations with high viscosity for controlled cleaning features. Here, we report our recent advancements in the design of halloysite-based nanomaterials for the controlled cleaning of solid surfaces as well as for the treatment of lignocellulosic historical objects.

### Controlled Surface Cleaning

In conservation science, surface cleaning is an intricate step due to very restrictive prescriptions. In particular, the process, besides being efficient, should not damage the surface or show the sign of time (aging) and does not contaminate the surface with residues that can induce damage to artwork. Due to the remarkable physicochemical property requirement, complex fluids and colloidal systems have been designed and proposed for these purposes.^65−68^

Within surface cleaning purposes, halloysite nanotubes are also promising, and they were tested in the following different formulations: (1) inorganic micelles and emulsions and (2) Pickering emulsions.

#### Inorganic Micelles and Emulsions

Due to the chemical and electrical properties of halloysite surfaces, clay nanotubes can be selectively modified by using ionic surfactants.^21,24^ Specifically, surfactants with a negative headgroup represent proper compounds for the functionalization of the halloysite inner surface, which is positively charged within a pH range between 2 and 8. The anionic surfactant/halloysite hybrids can be considered to be inorganic micelles with the different hydrophilic/hydrophobic character of their surfaces. Specifically, their shell preserves the hydrophilic behavior of pure halloysite, while their cavity becomes hydrophobic as a consequence of the alkyl chains of the surfactants. Halloysite-based inorganic micelles were obtained through sodium alkanoates,^21,24,29^ sodium perfluoroalkanoates,^23^ and sodium dodecyl sulfate.^24^ As a general approach, the hydrophobization of a halloysite cavity can be achieved by the following preparation steps: (1) Mixing of halloysite powders with saturated aqueous solutions of the surfactants. Stable dispersions are obtained after magnetically stirring for 48 h at 20 °C. (2) Centrifugation of the aqueous dispersions in order to separate the functionalized nanotubes by the aqueous phase. (3) Washing cycles of the functionalized nanotubes by water to remove the free surfactant fraction.

It was demonstrated^21^ that sodium alkanoate/halloysite nanotube composites are efficient in the adsorption of both aliphatic (*n*-decane) and aromatic (toluene) hydrocarbons, which are trapped in the hydrophobically modified lumen. This capacity is perspective for surface cleaning applications. Interestingly, the entrapment of hydrocarbons is reached with surfactant concentrations far below their critical micellar concentrations reducing the risks of residuals left on the cleaned surface.

**Figure 3:**
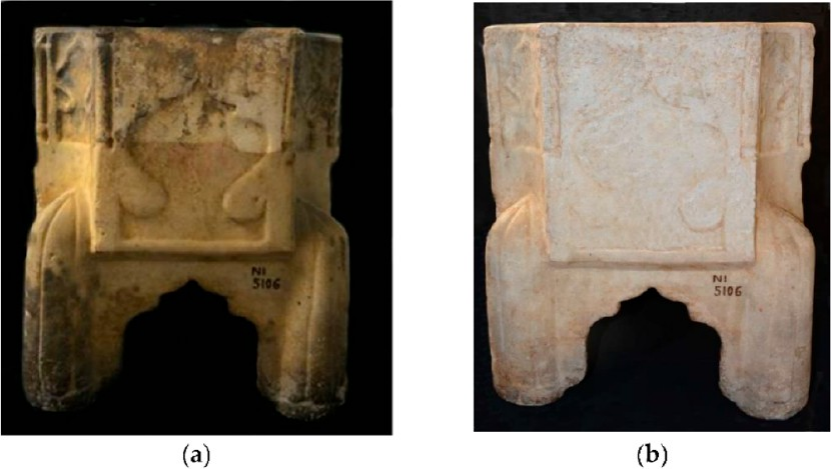
Photographs of the marble Kilga artifact (from the Sicilian Regional Museum) (a) before and (b) after surface cleaning treatment with the oil-in-water emulsion based on SDS/halloysite inorganic micelles and tetradecane. Adapted with permission from ref (69).

Halloysite nanotubes modified with sodium dodecyl sulfate (SDS) were investigated for the preparation of emulsions, which were successfully employed for the cleaning of a marble sculpture.^69^ For this purpose, tetradecane was selected as the oil phase. Figure 3 highlights that the proposed protocol is appropriate for the controlled cleaning of a marble artifact. In addition, FT-IR analyses of the cleaned surface evidenced that no residuals are present in the treated artwork.

Inorganic micelles based on sodium tetradecanoate and halloysite exhibited a relevant encapsulation ability toward tetradecane. The dispersion of the modified nanotubes within the chitosan matrix allowed us to fabricate a biofilm suitable for the dry cleaning of quart surfaces.^70^

##### Pickering Emulsion

**Figure 4:**
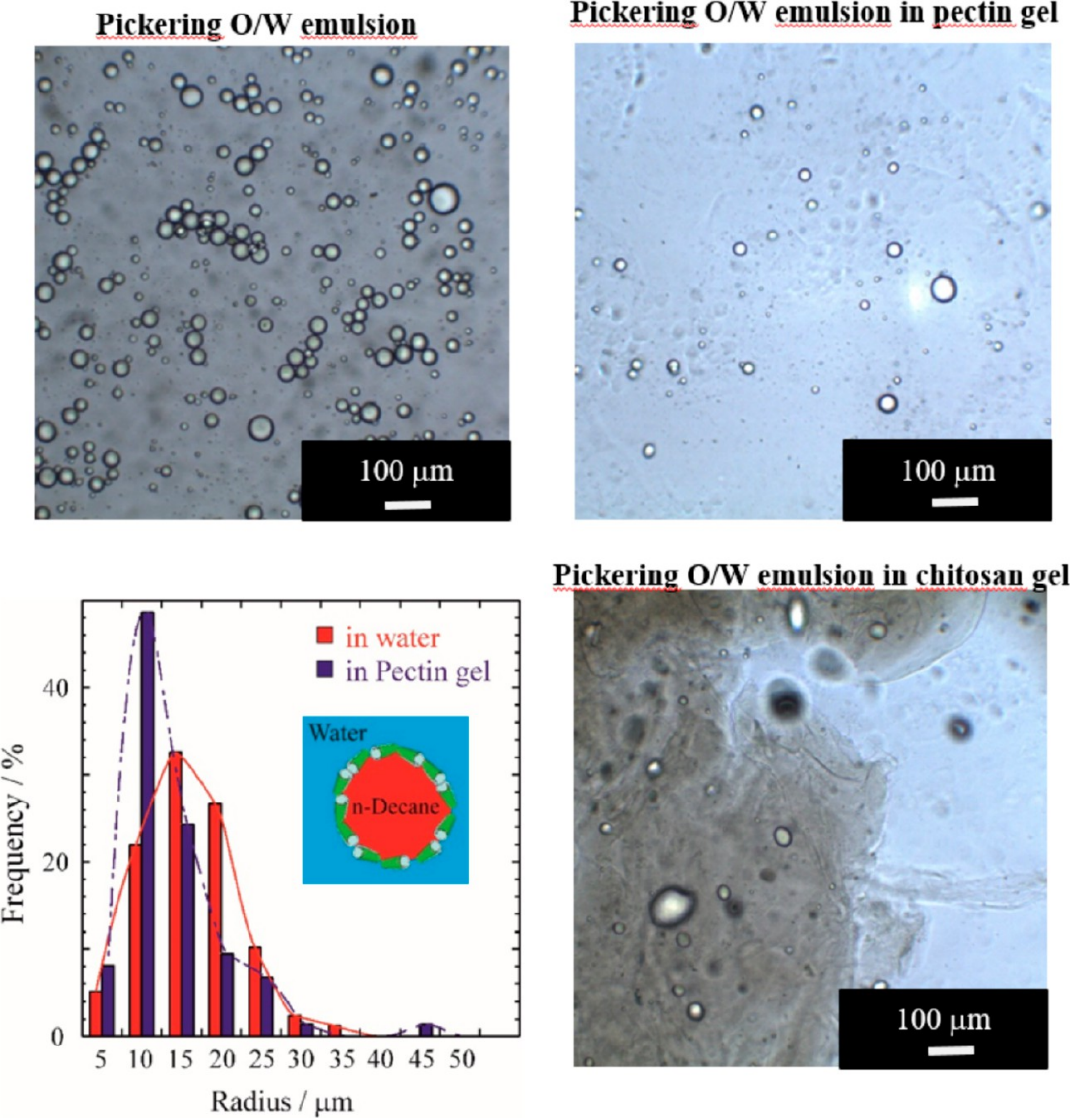
Optical images of Pickering emulsions and size distribution for oil droplets. Adapted with permission from ref (73).

An alternative route to the oil stabilization in aqueous solvent is represented by the Pickering emulsions, which are based on solid particles with great colloidal stability as a consequence of their opposition to coalescence processes and Oswald ripening.^30^ The recent literature shows that halloysite nanotubes are appropriate nanoclays for the preparation of Pickering emulsions useful for oil spill remediation.^71−73^ As presented in Figure 4, *n*-decane droplets with a radius of between 20 and 40 μm were successfully stabilized in a 1 wt % halloysite aqueous dispersion. Within conservation applications, the *n*-decane/halloysite Pickering emulsions were dispersed in a gel phase based on biopolymers (pectin and chitosan) largely employed as thickening agents. It was observed that the oil droplets are uniformly distributed in pectin gel (Figure 4). In contrast, a phase separation was detected within the chitosan matrix (Figure 4).

Moreover, the effect of biopolymers on the stability of the Pickering emulsion was thermodynamically investigated by the determination of the detaching free-energy change (Δ*G*_d_) for the nanotubes at the *n*-decane/water interface. The addition of both biopolymers induced a Δ*G*_d_ increase highlighting the enhanced affinity of the nanotubes toward the oil/water interface. Specifically, Δ*G*_d_ = 7.0 × 10^4^*kT* was estimated for the Pickering emulsion in water, while Δ*G*_d_ = 27 × 10^4^ and 125 × 10^4^*kT* were calculated in the presence of chitosan and pectin, respectively. These results evidenced that pectin is more efficient in the stabilization of halloysite at the oil/water interface. Namely, pectin is proper for the preparation of stable (1 month at least) Pickering emulsions based on halloysite and *n*-decane as the oil phase.

**Figure 5:**
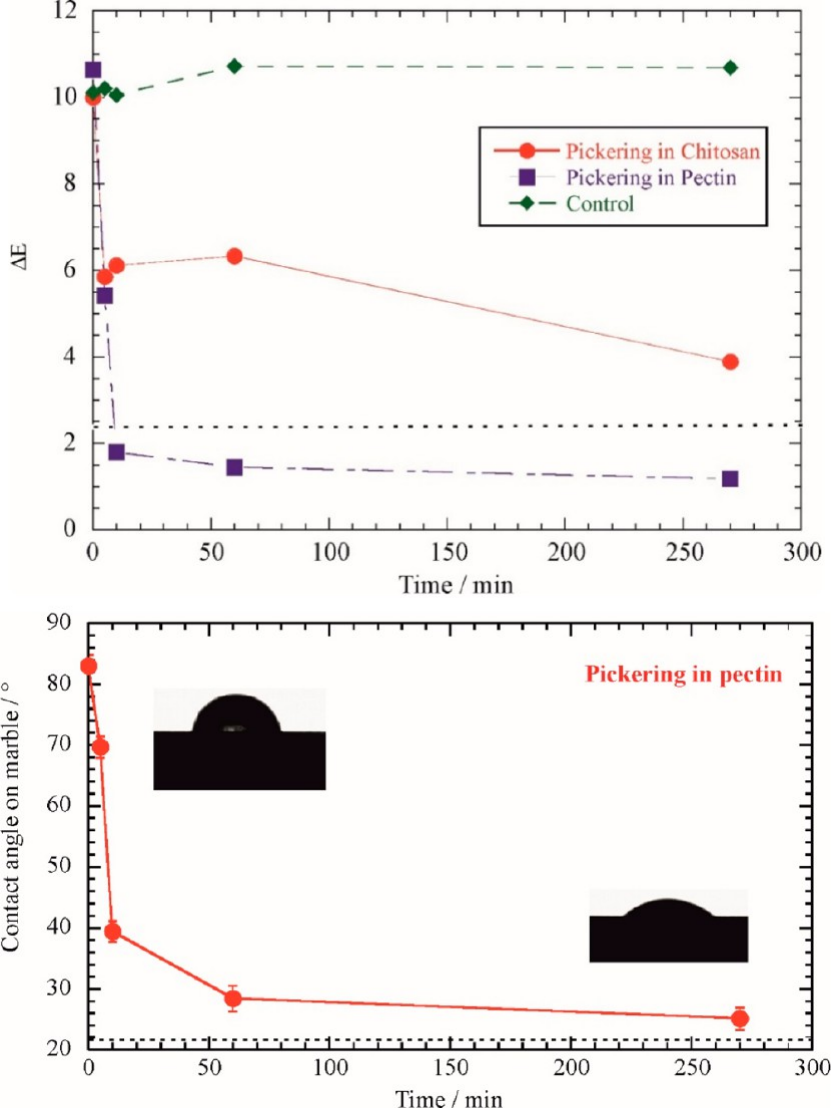
(Top) Effect of gel application time on the colorimetric parameter (Δ*E* on the CIE L*a*b scale) and (bottom) the water contact angle for marble surfaces during cleaning tests with Pickering emulsions in biopolymers gels. Dashed lines represent the thresholds for polished marble surface. Adapted with permission from ref (73).

Pickering emulsions in biopolymer gels were tested as cleaners for marble samples with a wax layer (thickness of ca. 100 μm) on their surfaces. It should be noted that wax was extensively employed for the protection of marble artworks, and its removal is crucial to developing suitable protocols for the restoration of cultural heritage. We explored the influence of the gel application time on the wax removal efficiency by measuring the colorimeter parameters as well as the initial water contact angle (Figure 5).

**Figure 6:**
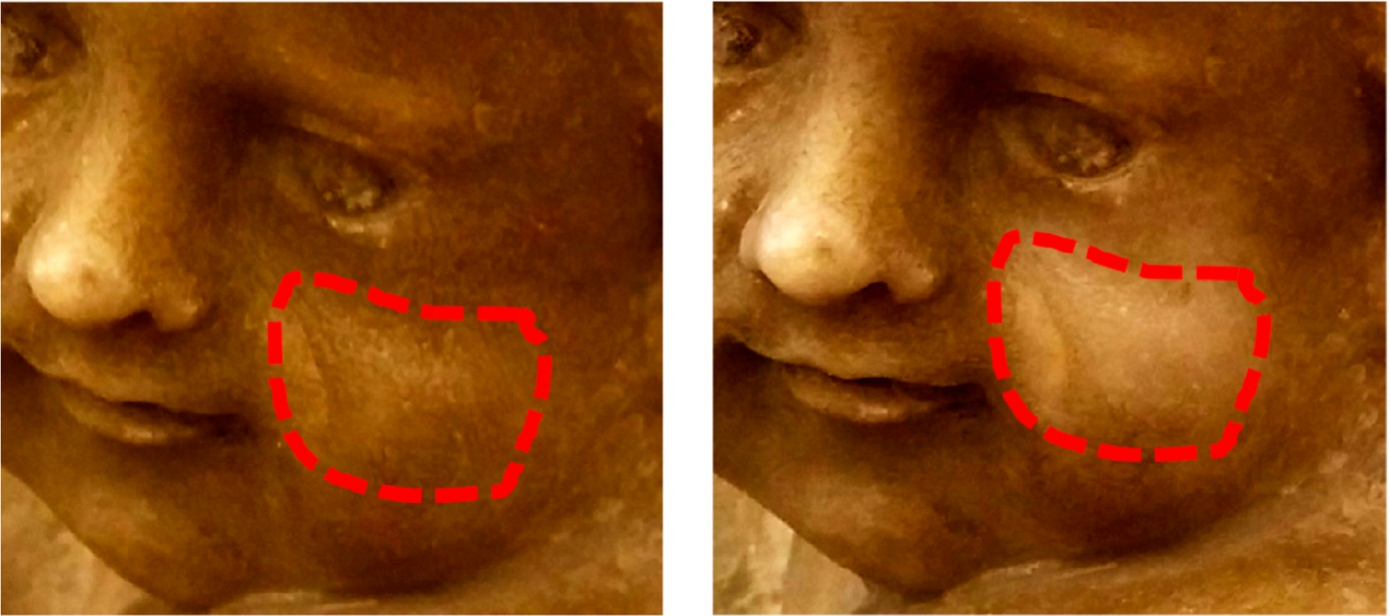
Photographs of the cherubs of the funeral monument of Placido Caruso (left) before and (right) after the treatment for 10 min with the Pickering in pectin gel. The cleaning area is bordered by the red dashed line. The monument is situated in Polizzi Generosa, Italy. Adapted with permission from ref (73).

Compared to the treatment with chitosan, the application of pectin gel generated a more significant variation of the colorimetric parameter (Δ*E*) highlighting a stronger cleaning efficacy on the marble surface. It should be noted that a color difference expressed on the CIE L*a*b* scale by DE is negligible to the human eyes if it is smaller than ca. 2.3. This finding agrees with the different structural and thermodynamic characteristics of the Pickering emulsions in biopolymer gel phases. Figure 5 shows that the initial water contact angle of marble decreases with the application time of the Pickering emulsion in pectin gel, indicating that the surface assumes a more hydrophilic character. Accordingly, we can state that the treatment was effective in the removal of the hydrophobic wax layer from the marble surface. In particular, we observed that the gel application for 50 min lowered the initial water contact angle from ca. 85° to ca. 25°, which indicates that hydrophobic compounds are not present on the marble surface. Additionally, cleaning tests with a Pickering emulsion in pectin gel were conducted on real artwork. As shown in Figure 6, a whitening of the sculpture was detected after 10 min of application, evidencing that the Pickering pectin gel could be used as a cleaner for marble surfaces.

## Conservation of Waterlogged Archeological Woods

Among the lignocellulosic artworks, waterlogged archeological woods have recently attracted growing interest by restorers and scientists.^74−78^ Wooden samples from ancient shipwrecks possess a high degree of porosity (up to 90% in volume), which causes structural deterioration and poor mechanical resistance. On the basis of these considerations, conservation protocols of waterlogged archeological woods are mostly aimed at filling the wooden pores. Poly(ethylene)glycols (PEGs) with variable molecular weight are generally employed as consolidants in the traditional treatments of archeological woods, and this approach was used for the consolidation of woods from Vasa (Sweden)^79^ and Batavia (Western Australia).^80^ Currently, PEGs are no longer considered suitable consolidants for woods as a consequence of the degradation of their −OH end groups that generate the formation of formic acid favoring the deterioration of the wooden structures. In this regard, calcium hydroxide nanoparticles^81−84^ were proposed as consolidants with deacidifying action, which prevent the acidic degradation of the wooden structure.

In our research, we developed innovative conservation protocols for waterlogged archeological woods by using an immersion method within dispersions containing halloysite nanotubes and sustainable polymers, including beeswax and colophony. In addition, a novel consolidant system based on PEG 1500 and halloysite nanotubes filled with calcium hydroxide was proposed for the simultaneous consolidation and deacidification of archeological woods. The proposed conservation protocols are discussed in the following paragraphs.

### Green Composites for Wood Consolidation: Beeswax/Halloysite and Colophony/Halloysite

**Figure 7:**
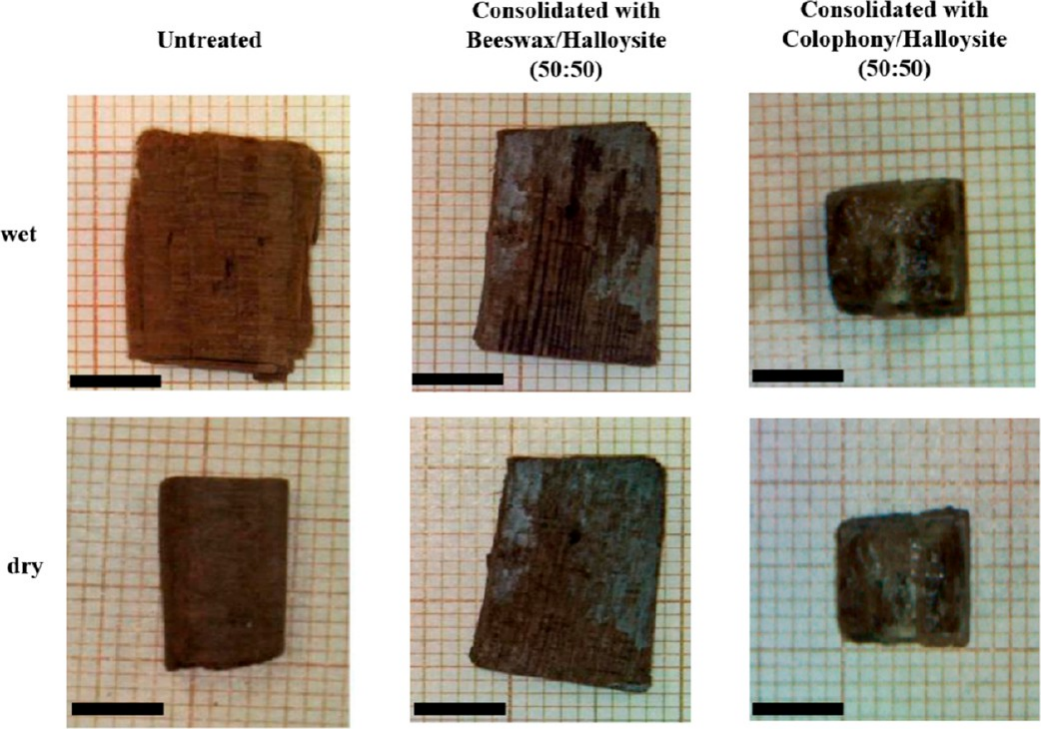
Optical images of untreated and treated woods. Bars are 0.5 cm. Adapted with permission from refs (85) and (86).

Green composite materials based on halloysite nanotubes and sustainable polymers (beeswax and colophony) were revealed as efficient consolidants for waterlogged archeological woods.^85^ The filling of the wooden pores was achieved by the wood immersion for 3 days within polymer/halloysite suspensions in acetone. It should be noted the dispersions were kept under magnetic stirring to facilitate the penetration of both polymer and halloysite into the wooden structure. The consolidation efficiency was estimated through the shrinkage volume (Δ*V*) of the archeological woods upon drying. It should be noted that the pore volume for a typical waterlogged archeological wood can be up to 90%, meaning that upon drying the cavities (representing most of the sample volume) become empty and the structure collapses, showing a strong volume contraction. Figure 7 displays the shrinking induced by drying for the wooden sample without and after consolidation treatment with halloysite-based mixtures.

As shown in Table 1, we detected that Δ*V* decreased after the immersion treatment, in agreement with the reduction of the wood porosity.

**Table 1:** Shrinkage Volume Results for Wood upon Drying

consolidant	shrinkage volume/%
no consolidation	40.6
beeswax	11.6
beeeswax/halloysite (70:30)^a^	6.2
colophony	18.1
colophony/halloysite (80/20)^a^	13.2

a The mass percentage compositions in the composite consolidants are reported.

We observed that the presence of halloysite in the conservation protocol enhanced the consolidation efficiency of both beeswax and colophony, with the Δ*V* values for woods consolidated by polymer/halloysite composites being significantly lower with respect to those related to the wooden samples treated by pure polymers. Specifically, the halloysite addition improved the consolidation efficiency by 46 and 27% for beeswax and colophony, respectively.

The consolidation efficiency results were correlated to the specific viscoelastic and thermal properties of the polymer/halloysite hybrids. As concerns beeswax, the presence of halloysite preserved the elastic component of the consolidant during polymer melting.^86^ On the other hand, the nanotubes reduced the heat capacity change for the glass transition of colophony as a consequence of the polymer adsorption onto halloysite surfaces.^85^

### PEG1500/Halloysite Filled with Calcium Hydroxide: Consolidation and Deacidification of Archeological Woods

**Figure 8:**
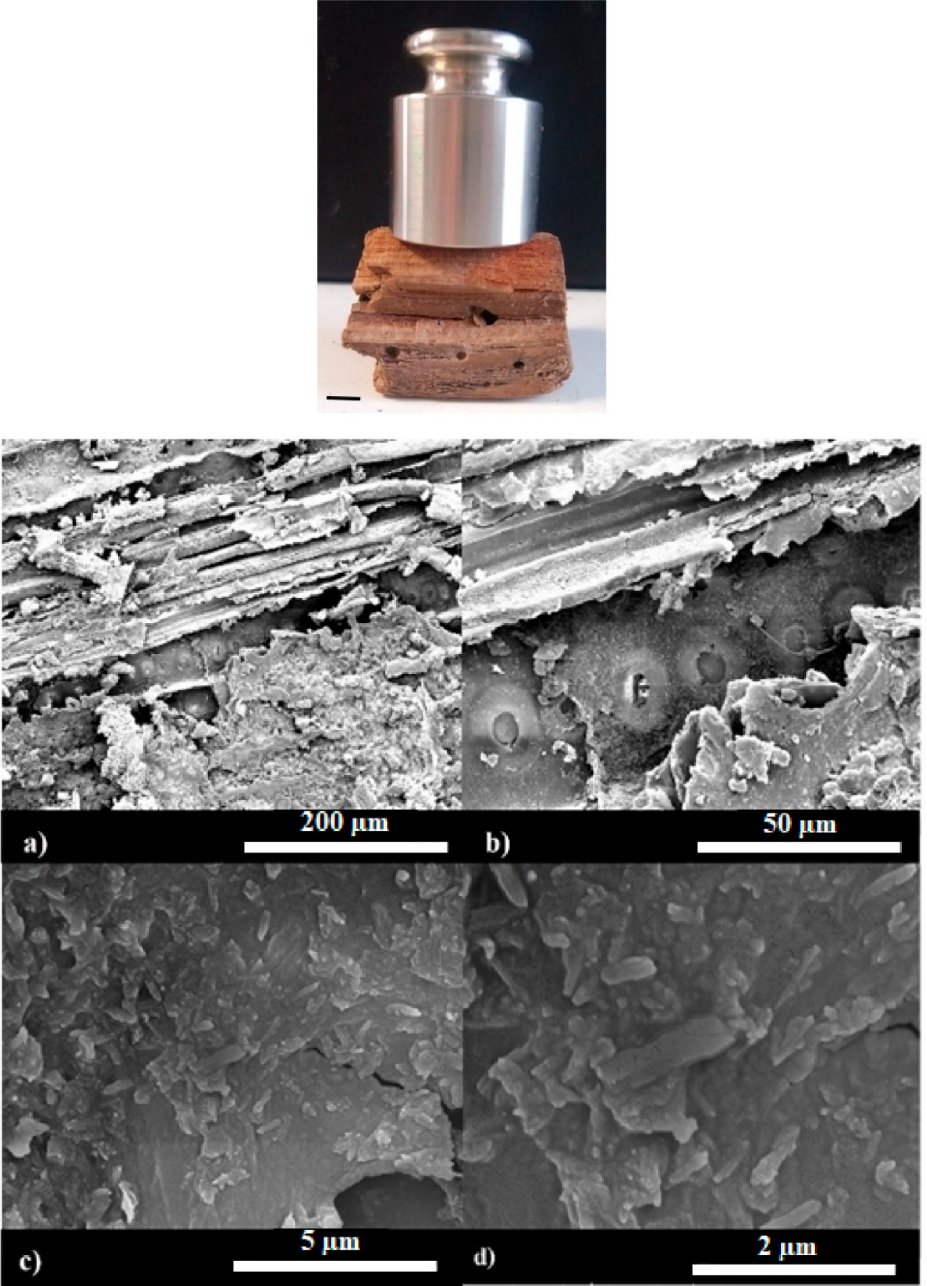
(Top) Optical photograph in which a mass of 100 g is placed on the top of the consolidated wood. The scale bar is 500 mm. (Bottom) Scanning electron images of waterlogged archeological wood at different magnifications (a–d). The percentage mass composition of the consolidated composite was set at 80:20 for PEG 1500/halloysite-Ca(OH)_2_. Adapted with permission from ref (87).

Recently, we proposed a novel procedure for the simultaneous consolidation and deadification actions toward waterlogged archeological woods through aqueous dispersions of PEG 1500 and halloysite nanotubes loaded with calcium hydroxide.^87^ Specifically, the PEG 1500 concentration (70 wt %) in the consolidant mixture was fixed, while the loaded halloysite composition was systematically changed. As previously described for beeswax/halloysite and colophony/halloysite systems,^85,86^ the wood immersion method for 3 days under magnetic stirring was employed. The filling of calcium hydroxide within halloysite was conducted by vacuum pumping in/out cycles as reported elsewhere for the encapsulation of active molecules^63^ inside the lumen of the nanotubes. On the basis of thermogravimetric analyses and assuming the rule of mixtures,^87^ we determined that the loaded amount of calcium hydroxide is 4.1 wt %. Figure 8 shows the optical and scanning electron images of wood consolidated by PEG 1500/halloysite-Ca(OH)_2_ (percentage mass composition 80:20).

**Figure 9:**
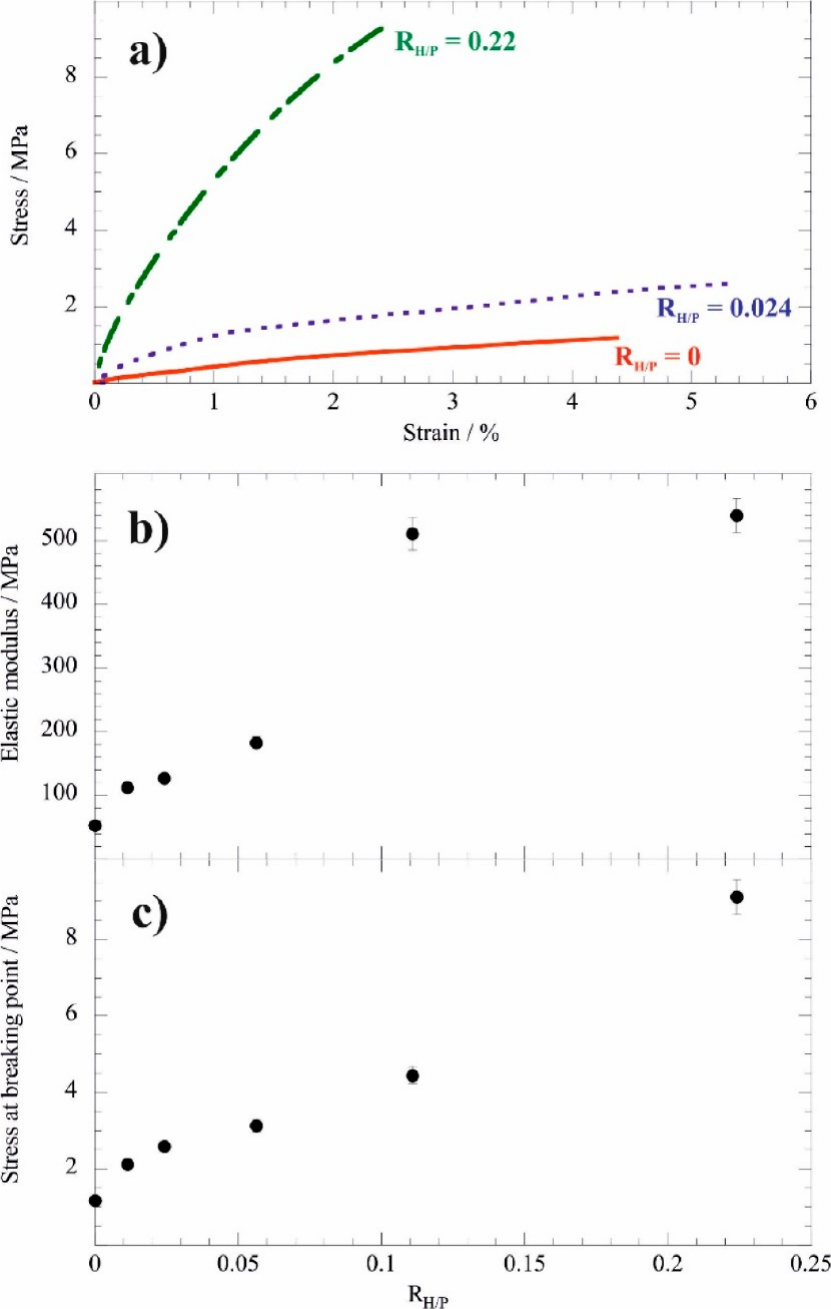
(a) Stress vs strain curves for consolidated wood samples. (b) The elastic modulus and (c) the stress at breaking point of the treated woods as functions of the consolidant composition expressed in mass ratio for halloysite-Ca(OH)_2_/PEG 1500 (*R*_H/P_). Adapted with permission from ref (87).

As evidenced by the optical photograph, the composite consolidant conferred robustness to the archeological wood, while SEM images highlighted that the wooden channel was successfully filled with the nanotubes, which are randomly dispersed within the PEG 1500 matrix (Figure 8). On this basis, we can state that the wood porosity was reduced by the consolidation treatment with PEG 1500/halloysite-Ca(OH)_2_ aqueous dispersion. According to the morphological investigations, the mechanical performances of the archeological woods were significantly improved by the penetration of the composite within the wooden structure. Figure 9a shows the stress vs strain curves obtained from flexural experiments conducted to woods treated by consolidant with variable composition (*R*_H/P_ represents the mass ratio between the Ca(OH)_2_-loaded halloysite and PEG 1500).

As flexural properties, the elastic modulus and the stress at the breaking point were determined from the analysis of the stress vs strain curves. Interestingly, the woods treated by the composite exhibited improved mechanical performance with respect to the wooden sample consolidated with pure polymer. Compared to the wood consolidated by neat PEG 1500, the elastic modulus of the sample treated by the composite with *R*_H/P_ = 0.22 is 1 order larger in agreement with its higher stiffness (Figure 9). Similarly, the stress at the breaking point was strongly enhanced (up to ca. 9 times) by the presence of halloysite-Ca(OH)_2_ in the consolidation protocol. Regarding the deacidification action of the consolidant, the effect of artificial aging on the lignin index (L.I.) of the wooden samples was investigated as it is correlated to the lignin content in the sample and therefore to the degradation state of the wood. The L.I. value was calculated from the IR spectra as the ratio between the peak intensity of the lignin group at 1511 cm^–1^ with the signals for aliphatic moieties (−CH_3_ groups) at 1375 cm^–1^. Artificial aging was conducted by the wood exposure to HNO_3_-saturated vapor for 3 days.

**Figure 10:**
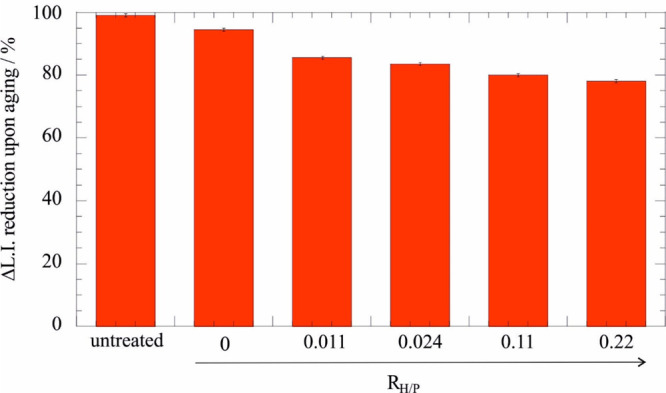
Lignin index reduction for untreated and treated wood samples exposed to HNO_3_ vapors for 3 days. Adapted with permission from ref (87).

As expected, a lignin index reduction (Δ_L.I._) was observed after the aging treatment because of the deterioration of the wooden structure (Figure 10). Namely, 100% of Δ_L.I._ reduction indicates the complete quantitative degradation of the lignin in the sample due to the acid degradation while 0% of Δ_L.I._ reduction would indicate that the lignin content is not altered by the aging protocol.

We estimated that the increase in the halloysite-Ca(OH)_2_ content in the consolidant mixture reduces the Δ_L.I._ values. Accordingly, we can state that the addition of loaded nanotubes improved the deacidification efficacy toward waterlogged archeological woods. The deacidifying ability of the consolidant is due to the Ca(OH)_2_ confined within the halloysite lumen. It should be noted that incorporating the Ca(OH)_2_ into the lumen retards the carbonatation reaction and prolonged the efficacy of the treatments.^88^

## Paper Treatment

Compared to other historical artifacts, paper and book goods typically require treatments capable of preserving from further degradation while keeping it possible, in some cases, to be accessible by the public. Over the centuries, paper has undergone a natural process of degradation that causes chemical and physical changes on it, although cellulose is a very stable material. In fact, paper made from cellulose alone is very resistant over time and does not turn toward degradation easily. On the other hand, the observed acidification process during paper aging has to be attributed to the substances added during the production processes such as pigments and binders.

The degradation of paper is mainly caused by hydrolytic and oxidative reactions. These processes lead to a worsening of the mechanical performance of the fibers due to the depolymerization of the material and therefore to a severe damage of the sheets of paper. There is no common method to be applied for deacidification as the process also depends on the type of paper being used. Traditionally, it is carried out by immersing the sheets for a precise period in a calcium bicarbonate and calcium hydroxide aqueous solution, which provide the paper with an alkaline reserve capable of counteracting the onset of other acid processes. This type of deacidification, by immersion, obviously requires the disassembly of the book, the dissolution of all of the bonds that assemble it, and the destruction of its unity and can have unpleasant consequences on paper due to the strong alkaline conditions. A more innovative method is based on a stable colloidal dispersion of calcium/magnesium hydroxide nanoparticles in an proper solvent medium.^77,84,89^ Once deposited on the cellulose fibers, these particles neutralize the acidity, and then reacting with atmospheric carbon dioxide, they form the calcium/magnesium carbonate which keeps the pH at 7.5–8 as an optimal value for paper storage and constitutes an alkaline reserve.^84,90^

**Figure 11:**
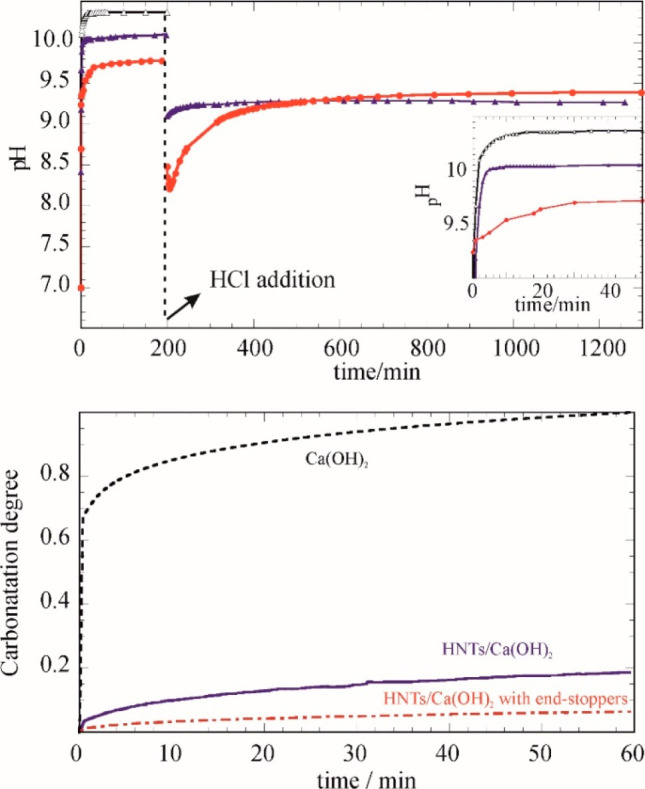
(Top) pH measurements in an aqueous dispersion before and after HCl solution addition as functions of time. Black, Ca(OH)_2_; purple, HNTs/Ca(OH)_2_; and red, HNTs/Ca(OH)_2_ with end stoppers. The inset reports an enlargement of the initial release. (Bottom) Degree of Ca(OH)_2_ carbonation in CO_2_ atmosphere. Adapted with permission from ref (88).

Recently, we proposed the use of Ca(OH)_2_ incorporated within the halloysite cavity to fabricate deacidifying filler for paper.^88^ The encapsulation of Ca(OH)_2_ within HNT was enhanced by the vacuum pumping protocol, and smart “end-stoppers” (made of Ca_3_(PO_4_)_2_) were designed to allow the stimuli-responsive release of Ca(OH)_2_ from the nanotube lumen.^21,88^ Ca(OH)_2_ carbonation was followed over time by thermogravimetry in a CO_2_ atmosphere. As Figure 11 shows, the calcium hydroxide confined in the halloysite lumen prevents and delays the carbonatation phenomenon with a further time extension in the presence of the Ca_3_(PO_4_)_2_ end-stoppers. Interestingly, in aqueous media this nanoarchitecture is responsive to the addition of HCl with a prompt buffer action.

The protection of paper under acidic conditions was monitored by tensile experiments on paper samples aged in an environment saturated by vapor from a nitric acidic solution. Comparing samples with same treatment time, one can argue that the Ca(OH)_2_/HNT system preserves the mechanical strength and neutral pH of the treated paper sample.

These findings not only represent a proof of concept but are open to the use of halloysite nanotubes as a nanocontainer for other active payloads to extend, for instance, the biocide or antioxidative stress response of a given treatment.

Finally, another relevant application in paper treatment is reported within flame-retardant action. With this in mind, the halloysite lumen was selectively modified with a fluorinated surfactant to generate a tubular cavity with gas storage capacity and therefore a flame-retardant effect.^23,91^ It should be noted that despite the very low content in fluorinated moieties (less than 1 wt % of the halloysite hybrid material), the burning rates slow down by a factor of 2 (for untreated paper it was ca. 5 mm s^–1^), and the combustion enthalpy was reduced from 6.02 to 4.88 kJ g^–1^ between untreated and treated paper, respectively.^91^

## Conclusions and Outlook

Halloysite nanotubes are sustainable natural nanoparticles that have a peculiar surface chemistry and nanocavity. These features open several routes to targeted modifications for the design of organic/inorganic hybrid architectures. Interesting physicochemical properties such as stimuli responsiveness, loading and controlled release of molecules, and mechanical and thermal reinforcing effects can be achieved. For these reasons, the application of halloysite nanotubes for the conservation of artwork is promising.

The clay used as thickeners for cleaning mixtures is a well-known application, notwithstanding modern chemistry allows nanoclay modification for the selective and controlled cleaning in some cases reducing the solvent mobility due to its incorporation in nano- or micropores. Therefore, the cleaning system should have high capillary forces/porosity.

Surface cleaning of solid substrates was achieved by filling biopolymers with inorganic micelles or Pickering emulsions based on halloysite nanotubes. Nanosponges and gels with halloysite were prepared and used for controlled cleaning applications. In consolidation and protection, the main perspectives are devoted to obtaining nanomaterials with smart performances and stimuli-responsive features. These characteristics are strategic in many aspects of the conservation of cultural heritage such as controlled cleaning or the development of smart protective coatings and active consolidants. The use of nanotubular clays put forward new sustainable strategies for removing the cost barrier typical of nanotechnologies. In the case of coating or protective additives, nanoclays are proposed as nanocontainers for active species to generate a time-extension in the efficacy of the treatment.
